# A pan-cancer analysis of DDR1 in prognostic signature and tumor immunity, drug resistance

**DOI:** 10.1038/s41598-023-27975-9

**Published:** 2023-04-08

**Authors:** Longfei Yang, Yuwei Zhang, Yifan Tang, Yang Wang, Peng Jiang, Fengping Liu, Ninghan Feng

**Affiliations:** 1 Medical School of Nantong University, 19 Qixiu Road, Nantong, 226001 Jiangsu China; 2 Department of Urology, Affiliated Wuxi No. 2 Hospital of Nanjing Medical University, 68 Zhongshan Road, Wuxi, 214002 Jiangsu China; 3 Wuxi School of Medicine, Jiangnan University, 1800 Lihu Avenue, Wuxi, 214028 Jiangsu China

**Keywords:** Oncogenes, Data mining, Computational biology and bioinformatics

## Abstract

Disk-like domain receptor 1 (DDR1) is a crucial regulator of pro-inflammatory mediators and matrix-degrading enzymes. Although mounting evidence supports a vital role for DDR1 in the tumorigenesis of some cancers, no pan-cancer analysis of DDR1 has been reported. Therefore, we aimed to explore the prognostic value of DDR1 in 33 cancer types and investigate its potential immune function. We used a range of bioinformatics approaches to explore the potential carcinogenic role of DDR1 in multiple cancers. We found that DDR1 was expressed at high levels in most cancers. DDR1 expression was positively or negatively associated with prognosis in different cancers. DDR1 expression was significantly associated with DNA methylation in 8 cancers, while there was a correlation between DDR1 expression and RNA methylation-related genes and mismatch repair gene in most cancers. Furthermore, DDR1 expression was significantly associated with microsatellite instability in 6 cancers and tumor mutation burden in 11 cancers. In addition, DDR1 expression was also significantly correlated with immune cell infiltration, tumor microenvironment, immune-related genes, and drug resistance in various cancers. In conclusion, DDR1 can serve as a potential therapeutic target and prognostic marker for various malignancies due to its vital role in tumorigenesis and tumor immunity.

## Introduction

The incidence and mortality of cancer have increased rapidly year by year, seriously endangering human health worldwide^1^. In recent years, tumor immunotherapy has emerged as a promising approach to cancer treatment, such as targeted PD-1/PD-L1^2^. However, cancer cells have also generated complex ways to escape from attacks on the immune system. For instance, mutation of β2MG could lead to HLA loss, failing some neoantigens to present on the cell surface. Consequently, patients will develop drug resistance to PD-1 due to toxic T cell reactions^3^. Furthermore, the economic burden of cancer has increased worldwide^4^. Therefore, there is an urgent need to find new diagnostic biomarkers and new targets for cancer therapy.

Disk-like domain receptor 1 (DDR1), which consists of a disk-like domain and an extracellular domain, is a tyrosine kinase receptor^5^. When activated by collagen, DDR1 triggers the activation of a series of downstream signaling pathways that induce the expression of pro-inflammatory mediators and matrix-degrading enzymes^6^. A previous study revealed that DDR1 inhibition could reduce neuropathological and inflammatory responses in neurodegenerative disease^7^. DDR1 participates in tumor progression by regulating cellular functions, including migration, cytokine secretion, and extracellular matrix homeostasis/remodeling. For example, DDR1 enhanced mammary tumor growth by regulating the production of interleukin-6 in mice^8^. Another study proved that DDR1 increased extracellular matrix remodeling through its ligand extracellular matrix proteins leading to liver metastasis in uveal melanoma^9^. Furthermore, the extracellular matrix (ECM) of the tumor microenvironment was enhanced by the extracellular domain of DDR1, which inhibited tumor T-cell infiltration and promoted tumor growth in a triple-negative breast cancer mouse model^10^. DDR1 promoted development in orthotopic glioblastoma mouse models and human ER-positive, HER2-negative breast cancer and reduced the efficacy of chemotherapy agents by activating the AKT signaling pathway in these tumor cells^11,12^. However, metformin could inhibit the expression of p-Akt and increase the expression of DDR1, decreasing cervical cancer progression^13^. Likewise, DDR1 is expressed at lower levels in clear cell renal cell carcinoma (ccRCC) and is related to shorter overall survival (OS)^14^. Therefore, comprehensive DDR1 analyses based on prognosis, tumor microenvironment, immune efficacy, and therapeutic efficacy in all cancer types are needed.

In the present study, we used the most up-to-date data from numerous databases, including The Cancer Genome Atlas (TCGA), Cancer Cell Line Encyclopedia (CCLE), Genotype Tissue-Expression (GTEx), and Gene Set Cancer Analysis (GSCA), to systematically analyze DDR1 expression levels and their relationship with prognosis in 33 types of cancer. We also assessed the relationship between DDR1 expression and DNA methylation, microsatellite instability (MSI), tumor mutation burden (TMB), and the tumor microenvironment (TME) in 33 types of cancer. We used co-expression analysis to analyze the relationship between DDR1 expression and RNA methylation-related genes, mismatch repair (MMR) gene, immune-related genes, and drug sensitivity in different cancers. Moreover, we investigated the biological function of DDR1 in tumors using single-cell sequencing database and enrichment analysis and verified the results with in vitro experiments with ccRCC, bladder cancer (BCa), and prostate cancer (PCa) cell lines. Our results suggest that DDR1 can serve as a prognostic factor for various cancers and play a vital role in tumor immunity by influencing MSI, TMB, and tumor-infiltrating immune cells.

## Methods

### Data collection

Gene expression, somatic mutations, and related clinical data from 33 types of cancer were downloaded from the UCSC Xena database (https://xenabrowser.net/datapages/), as well as RNA expression data of normal tissues of GTEx. The cell line expression matrix of RNA expression was downloaded from the CCLE database (https://portals.broadinstitute.org/ccle/).

### DDR1 methylation profile in pan-cancer based on GSCA

To evaluate the differential degree of methylation of DDR1 between tumor and corresponding normal samples, the correlation between DDR1 mRNA expression and DNA methylation in different cancer types was analyzed based on the GSCA database.

### Correlation of DDR1 expression with RNA methylation-related genes

RNA methylation modifications, including m6A (N6-methyladenosine), m5C (methyl-5-cytosine), and m1A (N1-methyladenosine), give rise to different outcomes that influence RNA functions^15^. Expression profile data from TCGA was used to evaluate the correlation between DDR1 and the levels of m6A-, m5C-, and m1A-related genes in different cancers, including Cbl proto-oncogene like 1 (CBLL1), WT1 associated protein (WTAP), methyltransferase 3 N6-adenosine-methyltransferase complex catalytic subunit (METTL3), METTL14, RNA binding motif protein 15 (RBM15), RBM15B, zinc finger CCCH-type containing 13 (ZC3H13), alkB homolog 5, RNA demethylase (ALKBH5), FTO alpha-ketoglutarate dependent dioxygenase (FTO), insulin like growth factor 2 mRNA binding protein 1 (IGF2BP1), YTH domain containing 1 (YTHDC1), YTHDC12, YTH N6-methyladenosine RNA binding protein 1 (YTHDF1), THDF2, YTHDF3, NOP2/Sun RNA methyltransferase family member 7 (NSUN7), NOP2/Sun RNA methyltransferase 2 (NSUN2), NSUN3, NSUN4, NSUN5, NSUN6, tRNA aspartic acid methyltransferase 1 (TRDMT1), DNA methyltransferase 1 (DNMT), DNMT3, DNA methyltransferase 3 alpha (DNMT3A), DNMT3B, NOP2 nucleolar protein (NOP2), tet methylcytosine dioxygenase 2 (TET2), tRNA methyltransferase 6 non-catalytic subunit (TRMT6), Aly/REF export factor (ALYREF), tRNA methyltransferase 61A (TRMT61A), TRMT61B, alkB homolog 1, histone H2A dioxygenase (ALKBH1), and alkB homolog 3, alpha-ketoglutarate dependent dioxygenase (ALKBH3).

### Analysis of the relationship between DDR1 and prognosis

Kaplan–Meier (KM) and univariate Cox regression analyses were performed to evaluate the OS, disease-specific survival (DSS), disease-free interval (DFI), and progression-free interval (PFI) of patients from the TCGA database. Univariate Cox regression analysis was conducted using the R packages “survival” and “forestplot” to assess the relationship between DDR1 expression and survival across cancers.

### Correlation of DDR1 expression with mismatch repair gene expression, microsatellite instability, and tumor mutation burden

TCGA expression profile data were used to assess the expression level of MMR gene expression, including mutL homolog 1 (MLH1), mutS homolog 2 (MSH2), MSH3, MSH4, MSH5, MSH6, PMS1 homolog 1, mismatch repair system component (PMS1), and PMS2 in different cancers and to evaluate the correlation between the levels and those of DDR1. MSI scores for samples of 33 cancer types were determined based on somatic mutation data downloaded from the TCGA database. Data of varscan2 variant aggregation and masking was downloaded from the UCSC Xena database, and a Perl script was used to calculate TMB scores. Spearman’s rank correlation coefficient was used to analyze the relationship between DDR1 expression and MMR gene, MSI, and TMB.

### Relationship between DDR1 expression and immunity

The relationship between DDR1 expression and TME was studied through the ESTIMATE algorithm. This algorithm was used to calculate immune and stromal scores for each tumor sample of 33 types of cancer. The relationship between DDR1 expression and these scores was evaluated according to the degree of immune infiltration using the R packages “estimate” and “limma”. We downloaded the immune cell infiltration score of TCGA from the CIBERSORT database (https://cibersortx.stanford.edu/) and used CIBERSORT to calculate relative scores for 22 immune cells in 33 cancers. Spearman’s rank correlation coefficient was used to analyze the correlation between DDR1 levels and each immune cell infiltration level in cancer. In addition, we explored the association between DDR1 expression and immune-related genes, which are chemokine, chemokine receptor, major histocompatibility complex (MHC), immunosuppressive, immunostimulatory, and immune checkpoint-related genes, in 33 types of cancer, and used the R package “limma”; the “reshape2” and “RColorBrewer” packages were used to visualize the results.

### Drug sensitivity analysis

To evaluate the relationship between drug sensitivity and DDR1 mRNA expression, we assessed drug screening data using the GSCA database, which integrated genomic data for over 10,000 samples for 33 cancer types from TCGA and data over 750 small-molecule drugs from Genomics of Drug Sensitivity in Cancer (GDSC) database.

### Single-cell sequencing data analysis

To validate the different functions of DDR1 in cancer cells at the single-cell level, we downloaded relevant data from the CancerSEA database and created a heatmap. Furthermore, the t-SNE diagrams of all individual cells were also obtained from CancerSEA.

### Gene set enrichment analysis

Gene set enrichment analysis (GSEA) was conducted to explore the biological functions of DDR1 across cancers (https://www.gsea-msigdb.org/gsea/downloads.jsp). Kyoto Encyclopedia of Genes and Genomes (KEGG) gene sets were downloaded from the KEGG website (www.kegg.jp/kegg/kegg1.html) and used the R packages “limma”, “org.Hs.eg.db”, “clusterProfiler”, and “enrichplot” were used to investigate the functions of DDR1.

### Cell culture

The human proximal tubular epithelial cell line HK-2 and human ccRCC cell lines 786-O and Caki-1, immortalized normal human urothelial cell line SV-HUC-1 and human bladder urothelial carcinoma cell lines T24 and 5637, and human normal prostatic epithelial cell line RWPE-1 and human PCa cell lines PC-3 and DU145 were all purchased from Procell Life Science & Technology Co., Ltd. (Wuhan, China). HK-2, Caki-1, T24, and DU145 cells were cultured in DMEM medium (Procell) supplemented with 10% fetal bovine serum (FBS) and 1% penicillin/streptomycin. Furthermore, 786-O and 5637 cells were cultured in RPMI 1640 medium (Procell) supplemented with 10% FBS and 1% penicillin/streptomycin. SV-HUC-1 and PC-3 cells were cultured in Ham’s F-12K medium (Procell) supplemented with 10% FBS and 1% penicillin/streptomycin. RWPE-1 cells were cultured in complete medium of human prostatic epithelial cells (Procell). All the cell lines were cultured in an incubator with 5% CO_2_ at 37 °C.

### Reverse transcription-quantitative polymerase chain reaction

Total RNA from the above cells and their corresponding normal cell lines was isolated using an RNA kit (TransGen Biotechnology, Beijing, China). RNA concentration was measured using NanoDrop 2000 (Thermo Fisher Scientific, Waltham, MA, USA). Next, cDNA was obtained using reverse transcription (RT). Finally, RT-quantitative polymerase chain reaction (qPCR) was conducted to investigate the expression of DDR1 using Green qPCR SuperMix (TransGen Biotechnology). The specific primers in our study were as follows: DDR1, F-5ʹ-CCGACTGGTTCGCTTCTACC-3ʹ, and R-5ʹ-CGGTGTAAGACAGGAGTCCATC-3ʹ; β-actin, F-5ʹ-GACGTGGACATCCGCAAAG-3ʹ, and R-5ʹ-CTGGAAGGTGGACAGCGAGG-3ʹ. Finally, the relative expression of DDR1 was calculated using the 2−ΔΔCT method.

### Statistical analysis

All the gene expression data were normalized by log2 transformation. The correlation analysis between the two variables used Spearman’s test or Pearson’s test; *P* < 0.05 was considered significant. Comparison of differences between adjacent normal and tumor tissues was performed using Wilcox test; *P* < 0.05 was indicated the statistical significance. The Kaplan–Meier curve and univariate Cox proportional hazard regression model were used for all survival analyses. All statistical analyses were processed by R software (Version 4.1.2).

## Results

### Differential expression of DDR1 between tumor and normal tissue samples

**Figure 1:**
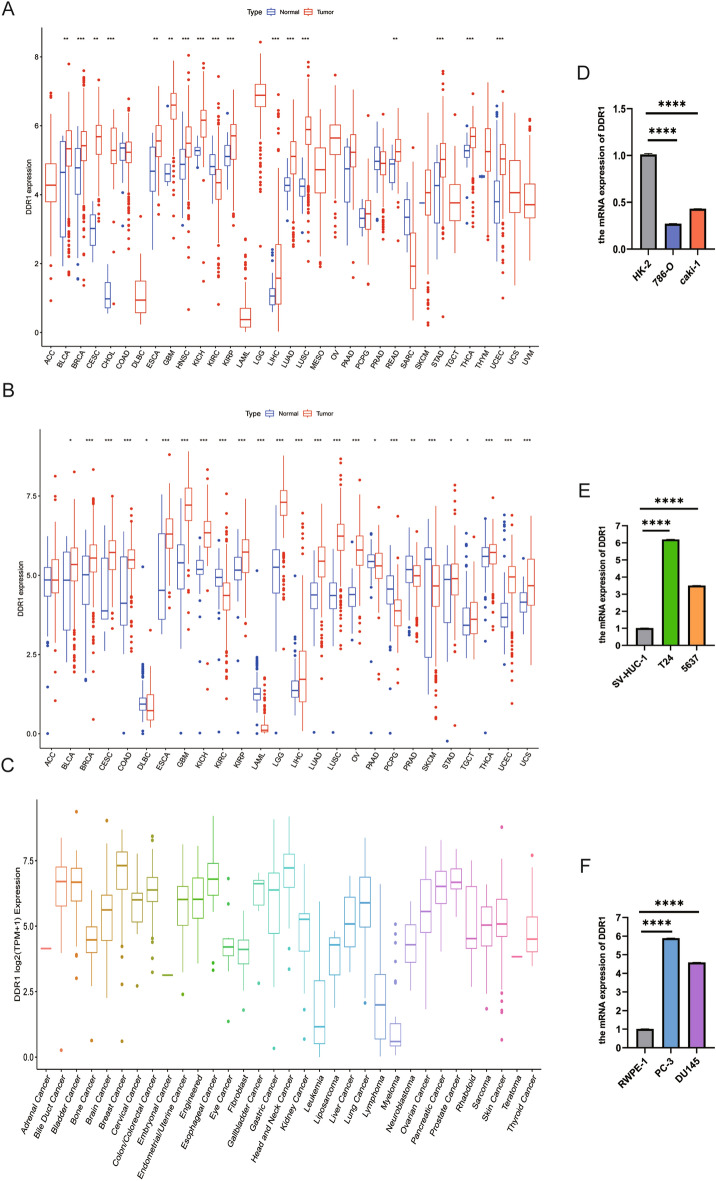
Differential expression of DDR1. (**A**) Comparison of DDR1 expression between tumor and normal samples in the TCGA database. (**B**) Comparison of DDR1 expression between tumor and normal samples in the TCGA and the GTEx databases. (**C**) DDR1 expression in tumor cell lines. *P < 0.05, **P < 0.01, ***P < 0.001. (**D–F**) The expression level of DDR1 was evaluated in clear cell renal cell carcinoma (ccRCC), bladder cancer (BCa), and prostate cancer (PCa) cells and their corresponding normal cells by qRT-PCR.

To better understand DDR1 expression levels in various cancer types, we first performed a pan-cancer analysis of 33 cancers in the TCGA database. Excluding cancers without corresponding normal samples, significant differences in DDR1 expression were found between tumor and normal tissues in 17 types of cancer. DDR1 is highly expressed in bladder urothelial carcinoma (BLCA), breast invasive carcinoma (BRCA), cervical squamous cell carcinoma and endocervical adenocarcinoma (CESC), cholangiocarcinoma (CHOL), esophageal carcinoma (ESCA), glioblastoma multiforme (GBM), head and neck squamous cell carcinoma (HNSC), kidney chromophobe (KICH), kidney renal papillary cell carcinoma (KIRP), liver hepatocellular carcinoma (LIHC), lung adenocarcinoma (LUAD), lung squamous cell carcinoma (LUSC), rectum adenocarcinoma (READ), stomach adenocarcinoma (STAD), thyroid carcinoma (THCA), and uterine corpus endometrial carcinoma (UCEC). In contrast, DDR1 was down-regulated in tumor tissues relative to normal tissues in kidney renal clear cell carcinoma (KIRC) (Fig. 1A, Supplementary Table S1). As the number of normal samples for some cancers in the TCGA database was limited, we used data from the GTEx database, which includes data on diverse tissues from healthy persons. Our results showed that the expression of DDR1 was highly different in 25 of the 26 cancers versus normal tissues when the TCGA and GTEx databases were combined. DDR1 was expressed at lower levels in lymphoid neoplasm diffuse large B-cell (DLBC), KIRC, acute myeloid leukemia (LAML), pancreatic adenocarcinoma (PAAD), pheochromocytoma and paraganglioma (PCPG), prostate adenocarcinoma (PRAD), and skin cutaneous melanoma (SKCM) but was expressed at higher levels in another 18 tumors (Fig. 1B, Supplementary Table S1). Moreover, we also investigated the expression of DDR1 in different cancer cell lines based on the CCLE database (Fig. 1C). In addition, as shown in Fig. 1D–F, a relatively low expression DDR1 was detected in 786-O and Caki-1 cells than in HK-2 cells. In contrast, DDR1 expression was highly in BCa and PCa cells than in their corresponding normal cell lines. Taken together, these data indicate that DDR1 expression is dysregulated in various cancers versus adjacent normal tissues and may serve as an oncogene or suppressor in these cancers.

### DDR1 is associated with expression levels of DNA methylation and RNA methylation-related genes across cancers

**Figure 2:**
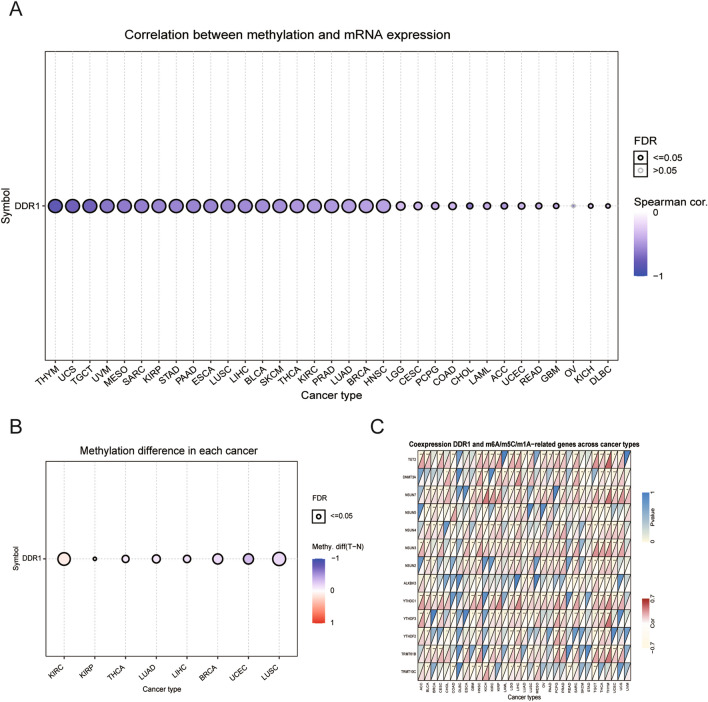
Association between DDR1 expression and DNA methylation and m6A-, m5C-, and m1A-related genes across cancers. (**A**) Correlation between methylation and mRNA expression levels of DDR1. (**B**) Methylation differences in DDR1 mRNA in different cancers. (**C**) Heatmap illustrating the relationship between DDR1 and m6A-, m5C-, and m1A-related genes. The top left triangle represents the P-value, and the bottom right triangle represents the correlation coefficient. *P < 0.05, **P < 0.01, ***P < 0.001.

DNA methylation alteration has been observed in various cancers and is considered a cause of carcinogenesis^16^. We analyzed the correlation between promoter methylation and mRNA expression of DDR1 using the GSCA database. Our data revealed a correlation between DDR1 expression and DNA methylation in 32 types of tumors (Fig. 2A, Supplementary Table S1). DDR1 expression and promoter methylation levels were significantly positive in BRCA, KIRP, LIHC, LUAD, LUSC, THCA, and UCEC, while negatively correlated in KIRC (Fig. 2B).

RNA methylation modifications, including m6A, m5C, and m1A, give rise to different outcomes influencing RNA functions and may contribute to tumorigenesis^15^. Likewise, in most tumors, DDR1 expression was positively correlated with YTHDC1, YTHDF2, TRMT61B, and DNMT3A, especially in LIHC, PCPG, and PRAD. As shown in Fig. 2C, NSUN2 and NSUN7 were positively correlated with DDR1 expression in BLCA, HNSC, LIHC, sarcoma (SARC), THCA, and thymoma (THYM). The results show that the altered epigenetic status of DDR1 may contribute to tumorigenesis.

### Prognostic significance of DDR1

**Figure 3:**
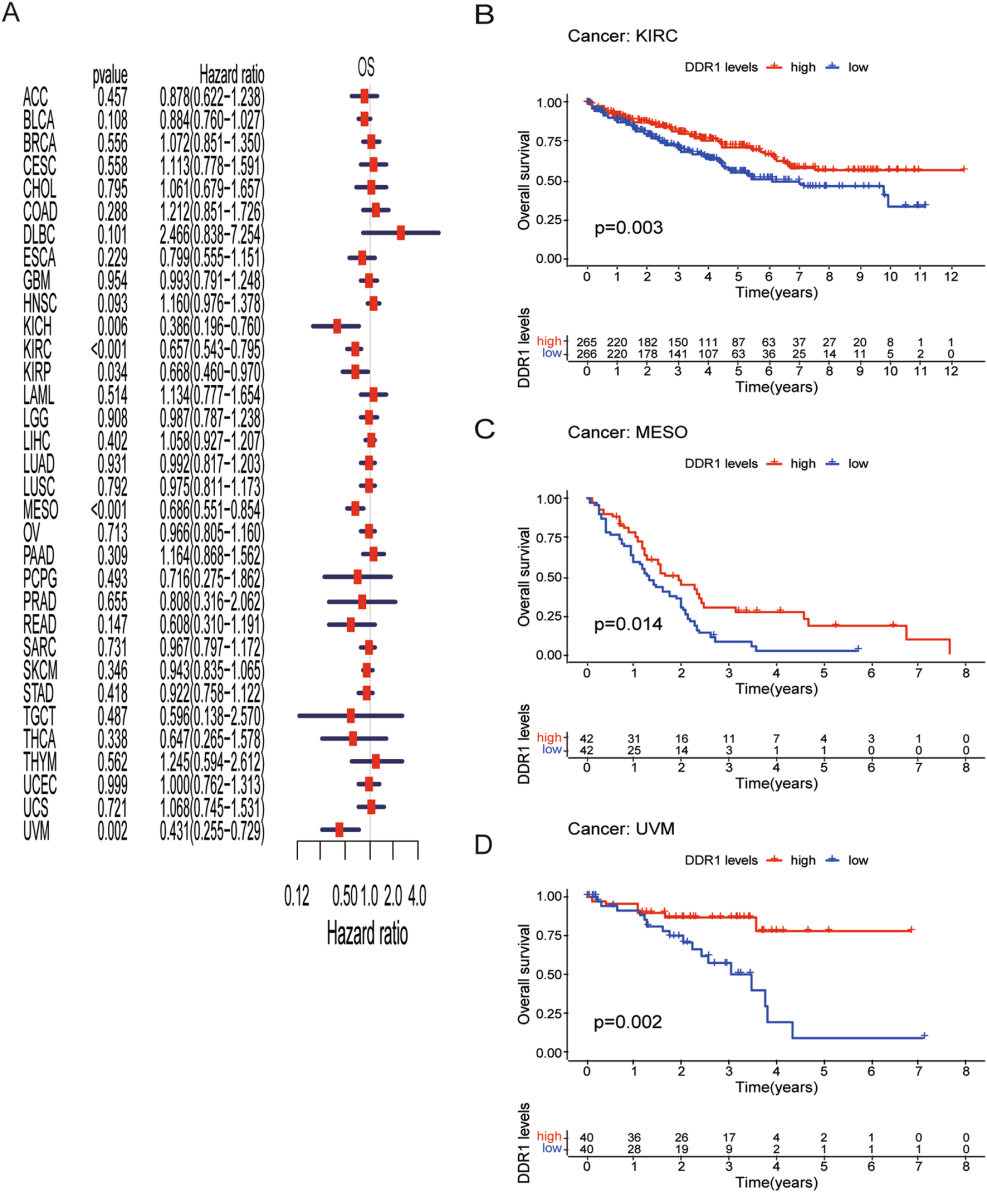
Association between DDR1 expression and overall survival (OS). (**A**) Forest plot shows the univariate Cox regression results for the association between DDR1 expression and OS in 33 types of tumors. (**B–D**) Kaplan–Meier analysis of the association between DDR1 expression and OS.

**Figure 4:**
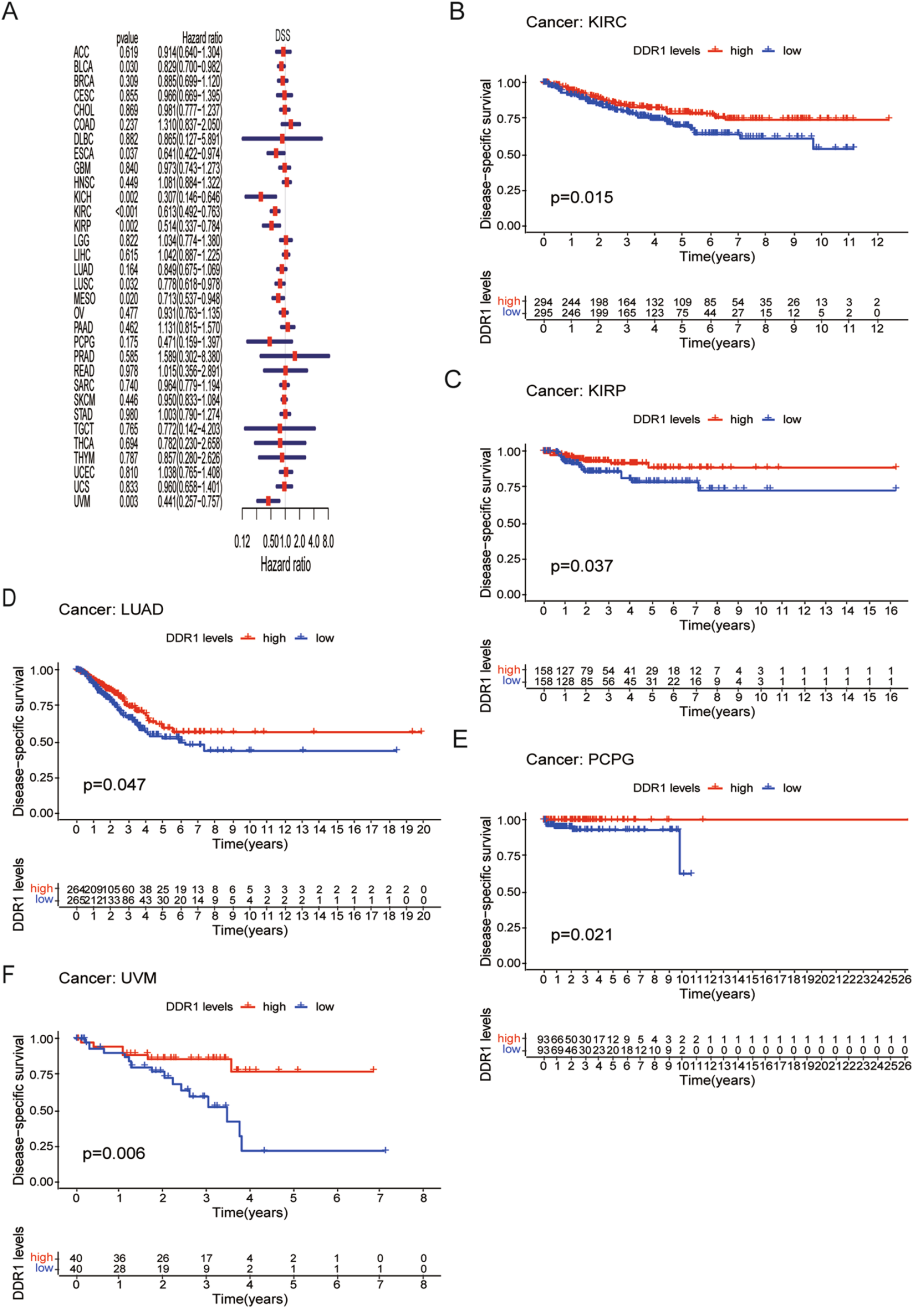
Association between DDR1 expression and disease-specific survival (DSS). (**A**) Forest plot shows the univariate Cox regression results for the association between DDR1 expression and DSS in 33 types of tumors. (**B–F**) Kaplan–Meier analysis of the association between DDR1 expression and DSS.

Next, we performed the prognostic value of DDR1 expression in pan-cancer, including OS, DSS, DFI, and PFI analyses based on the TCGA database. OS by Cox regression indicated that DDR1 was a protective factor for patients with KIRC, KIRP, KICH, MESO, and UVM (Fig. 3A). Similarly, the analysis of the KM OS revealed that DDR1 acts as a protective factor for patients with KIRC, mesothelioma (MESO), and uveal melanoma (UVM) (Fig. 3B–D). Since non-tumor-related factors may lead to death during follow-up, we analyzed the relationship between DDR1 expression and DSS in 33 cancer types. As shown in Fig. 4A, low DDR1 expression has corresponded with poor DSS in 8 types of cancer, including BLCA, ESCA, KICH, KIRC, KIRP, LUSC, MESO, and UVM. KM of DSS analysis revealed that high DDR1 expression corresponded with longer DSS in KIRC, KIRP, LUAD, PCPG, and UVM (Fig. 4B–F).

**Figure 5:**
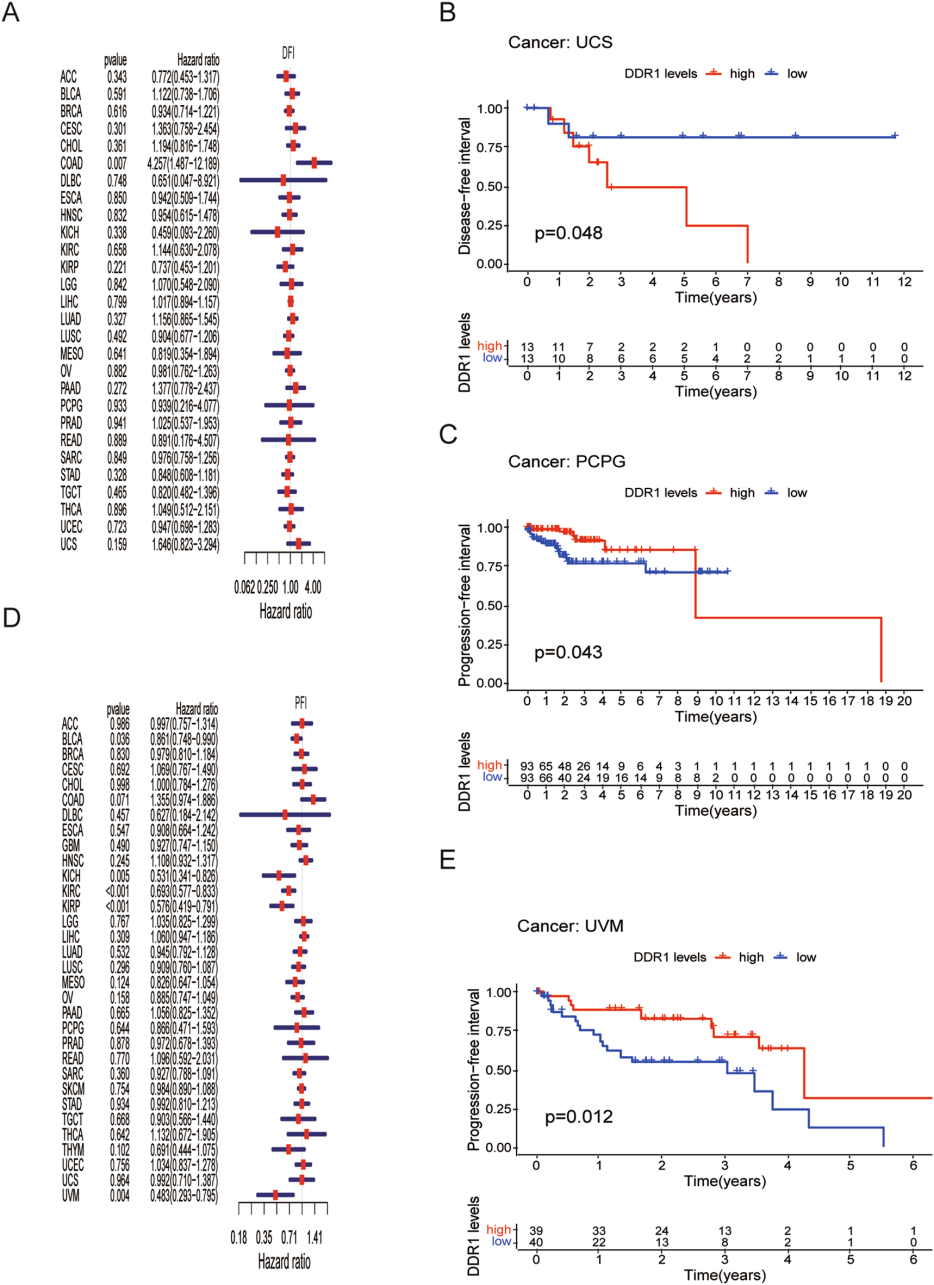
Association between DDR1 expression and disease-free interval (DFI) and progression-free interval (PFI). (**A, D**) Forest plot shows the univariate Cox regression results for the association between DDR1 expression and DFI and PFI in 33 types of tumors, respectively. (**B**) Kaplan–Meier analysis of the association between DDR1 expression and DFI. (**C, E**) Kaplan–Meier analysis of the association between DDR1 expression and PFI.

DFI was used to evaluate the outcome of radical surgery and generally determined the time from treatment to recurrence. Subsequently, we studied the relationship between DDR1 expression and DFI. Cox regression analysis revealed that high DDR1 expression was a risk factor for COAD patients, and KM analysis found that high DDR1 expression was associated with a poor prognosis in uterine carcinosarcoma (UCS) patients (Fig. 5A, B). In addition, we assessed the relationship between DDR1 expression and PFI, a measure of how well cancer responds to palliative care. The KM of PFI analysis showed that DDR1 was a protective factor for patients with PCPG and UVM, and Cox regression analysis showed that DDR1 was a protective factor in BLCA, KICH, KIRC, KIRP, and UVM (Fig. 5C–E). These data suggest that DDR1 expression is significantly associated with patient prognosis across multiple cancer types, especially in patients with KIRP, KIRC, and UVM.

### Correlation of DDR1 expression levels with mismatch repair gene expression, microsatellite instability, and tumor mutation burden

**Figure 6:**
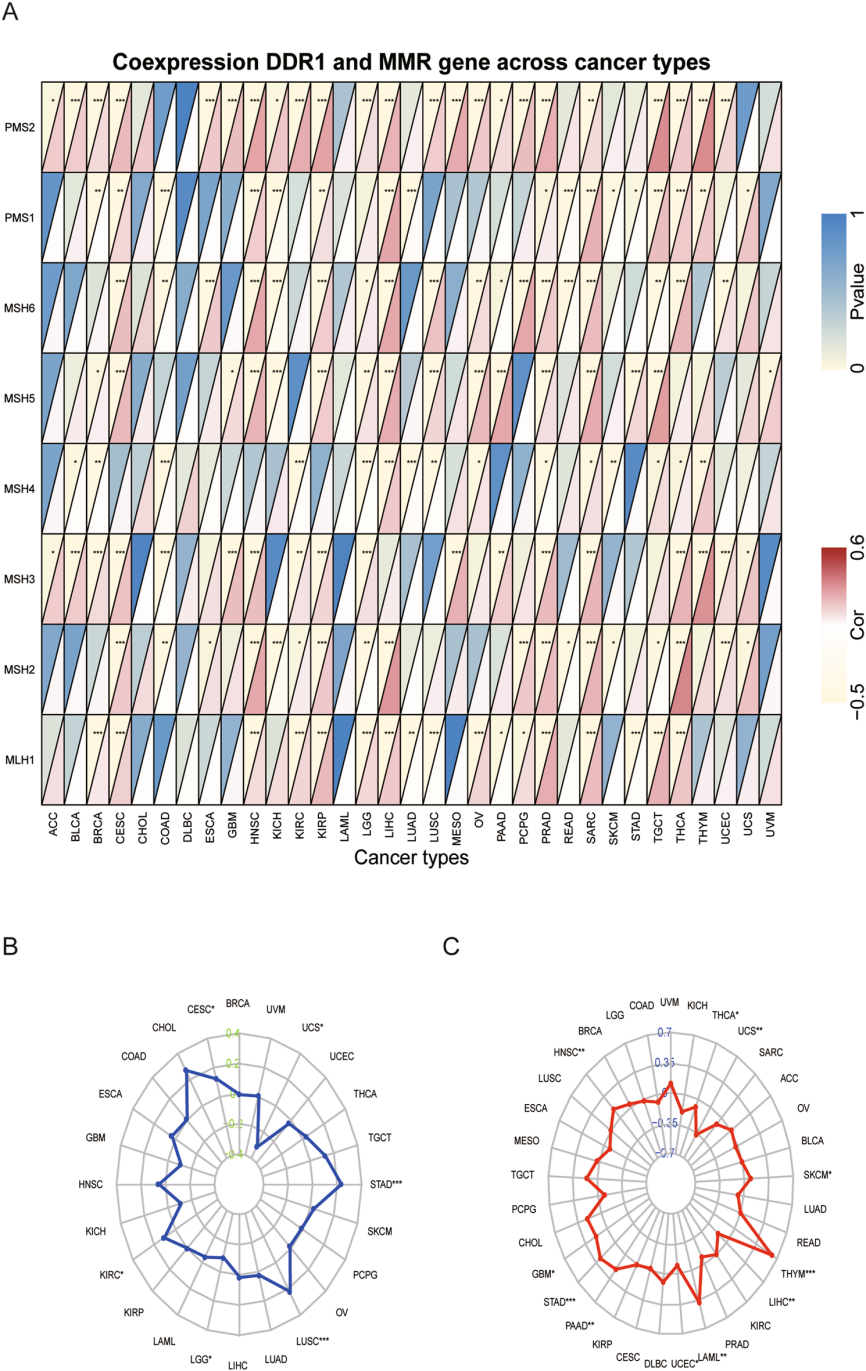
Association between DDR1 expression and mismatch repair (MMR) gene expression, microsatellite instability (MSI), and tumor mutational burden (TMB) across cancers. (**A**) Heatmap illustrating the relationship between DDR1 and MMR gene. The top left triangle represents the P-value, and the bottom right triangle represents the correlation coefficient. *P < 0.05, **P < 0.01, ***P < 0.001. (**B**) Correlation between DDR1 expression and MSI across cancers. (**C**) Correlation between DDR1 expression and TMB across cancers. The value of black represents the range, and the curves of blue and red represent the correlation coefficients.

Errors occur in DNA replication, and MMR gene can recognize and fix these errors^17^. Tumors with defects in the MMR system are vulnerable to mutations in microsatellites, causing high levels of MSI, which leads to the accumulation of mutation loads in cancer-related genes and the aggravation of TMB^18^. Hence, we analyzed the relationship between the expression of DDR1 and MMR gene, including MLH1, MSH2, MSH3, MSH4, MSH5, MSH6, PMS1, and PMS2. In most tumors (except COAD, DLBC, LAML, LUAD, and SKCM), MMR gene expression was significantly positively correlated with DDR1 expression (Fig. 6A, Supplementary Table S1). We also analyzed the relationship between the expression of DDR1 and MSI, and TMB. Our results showed that DDR1 was significantly correlated with MSI in CESC, KIRC, brain lower grade glioma (LGG), LUSC, STAD, and UCS (Fig. 6B). As shown in Fig. 6C, DDR1 expression was highly correlated with TMB in 11 types of tumors, including LAML, PAAD, STAD, and THYM. In summary, these results indicate that DDR1 may mediate tumorigenesis by regulating DNA damage.

### Relationship between DDR1 expression and tumor microenvironment

**Figure 7:**
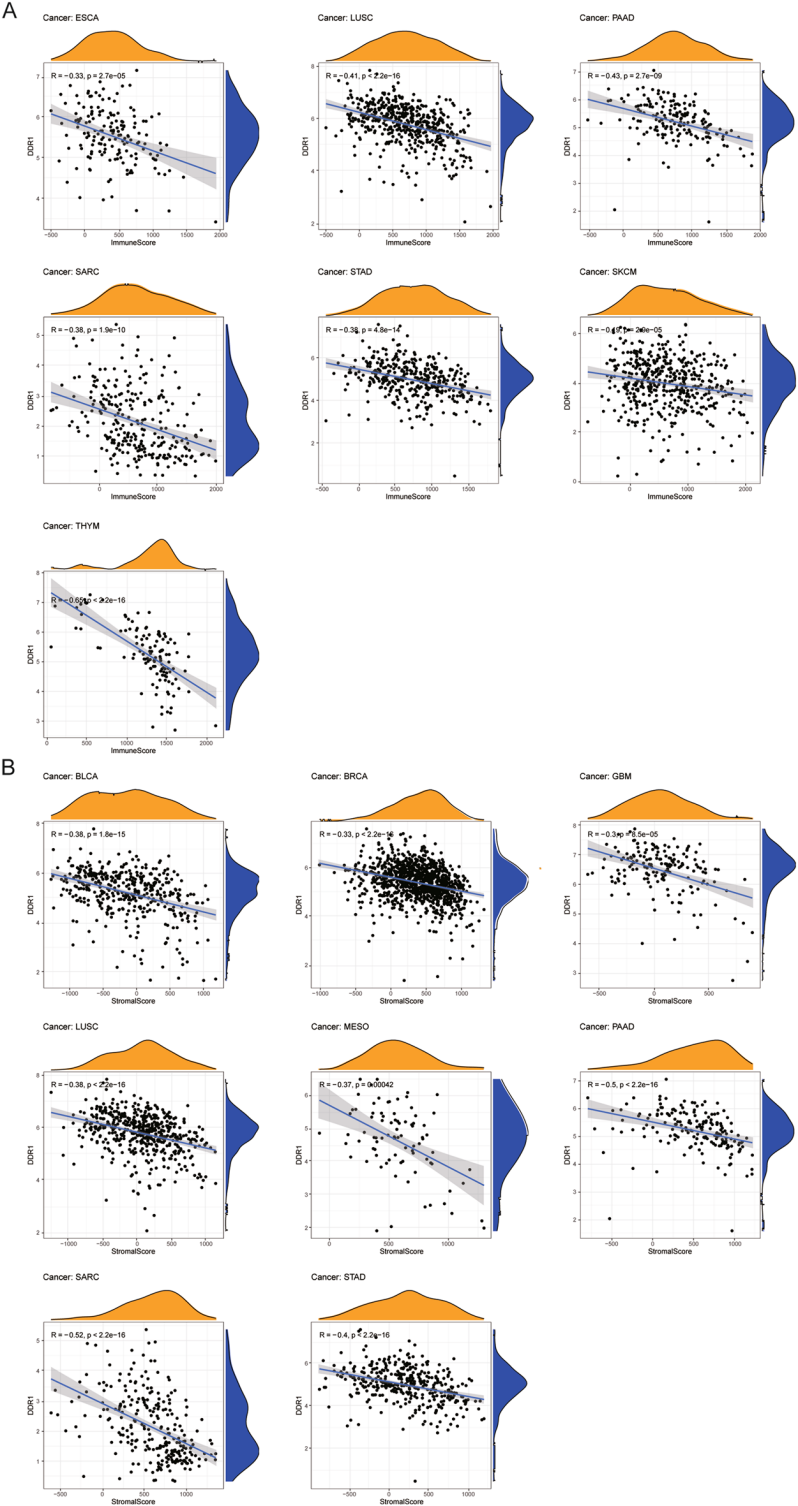
Eleven tumors with the highest correlation coefficients between DDR1 expression and the tumor microenvironment. (**A**) Correlation between DDR1 and immune scores in ESCA, LUSC, PAAD, SARC, STAD, SKCM, and THYM. (**B**) Correlation between DDR1 and stromal scores in BLCA, BRCA, GBM, LUSC, MESO, PAAD, SARC, and STAD.

An increasing number of reports have indicated that the tumor microenvironment plays a vital role in tumor occurrence and development^19^. Therefore, we investigated the pan-cancer relationship between TME and DDR1 expression, using the ESTIMATE algorithm to calculate the stromal and immune cell scores in 33 types of cancer (Supplementary Table S1). Our results revealed that DDR1 was negatively correlated with immune scores and stromal scores in 14 cancers (BLCA, BRCA, GBM, HNSC, KIRC, KIRP, LUAD, LUSC, PAAD, SARC, SKCM, STAD, THCA, and UCEC). The eleven tumors with the highest correlation coefficients are presented in Fig. 7; the results for other cancers are shown in Supplementary Fig. S1. These findings suggest that DDR1 may influence the immune tolerance of tumors by regulating TME.

### Relationship between DDR1 expression and levels of tumor immune cell infiltration

**Figure 8:**
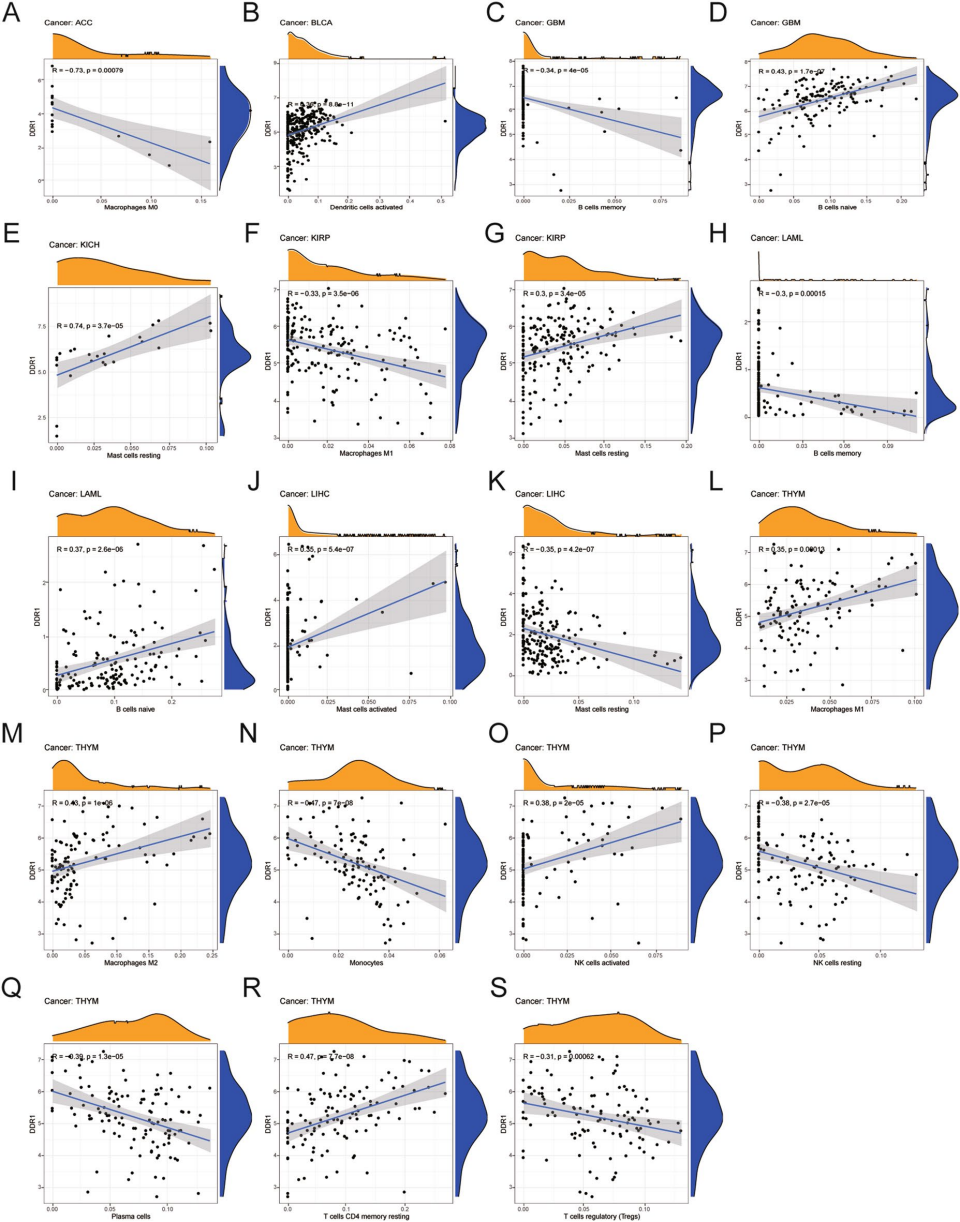
Relationship between DDR1 expression and tumor infiltration of different immune cells in the TCGA database. (**A–S**) Eight tumors with the highest correlation coefficients between DDR1 expression and immune-associated cell infiltration.

Next, we examined the relationship between DDR1 expression and the levels of infiltration of 22 immune-related cells. The results revealed that DDR1 was negatively correlated with the infiltration levels of M0 macrophages and activated CD4 T memory cells, while positively correlated with those of activated dendritic cells in TCGA pan-cancer datasets (Fig. 8, Supplementary Table S1). For example, DDR1 expression was negatively correlated with the levels of infiltration M0 macrophages in adrenocortical carcinoma (ACC), BLCA, BRCA, CESC, and LUSC. DDR1 expression was negatively correlated with activated CD4 T memory cells in COAD, LUAD, LUSC, and SARC, while positively related to those of activated dendritic cells in BLCA, CESC, LUAD, LUSC, PRAD, STAD, and UCEC. We also analyzed the correlation between DDR1 and B cells and NK cells in 33 types of cancer. DDR1 expression was positively correlated with naive B cells in GBM, HNSC, LAML, LUSC, and THCA, while negatively correlated in BLCA. In addition, DDR1 was positively correlated with activated NK cells in PAAD, STAD, and THYM but negatively correlated in KIRC. The eight tumors with the highest correlation coefficients between the degree of infiltration and DDR1 expression for each type of immune cell are presented in Fig. 8; the results for other cancers are shown in Supplementary Fig. S2. These results illustrate that DDR1 may contribute to cancer immune escape by mediating tumor immune cell infiltration.

### Relationship between DDR1 expression and levels of immune-related genes

**Figure 9:**
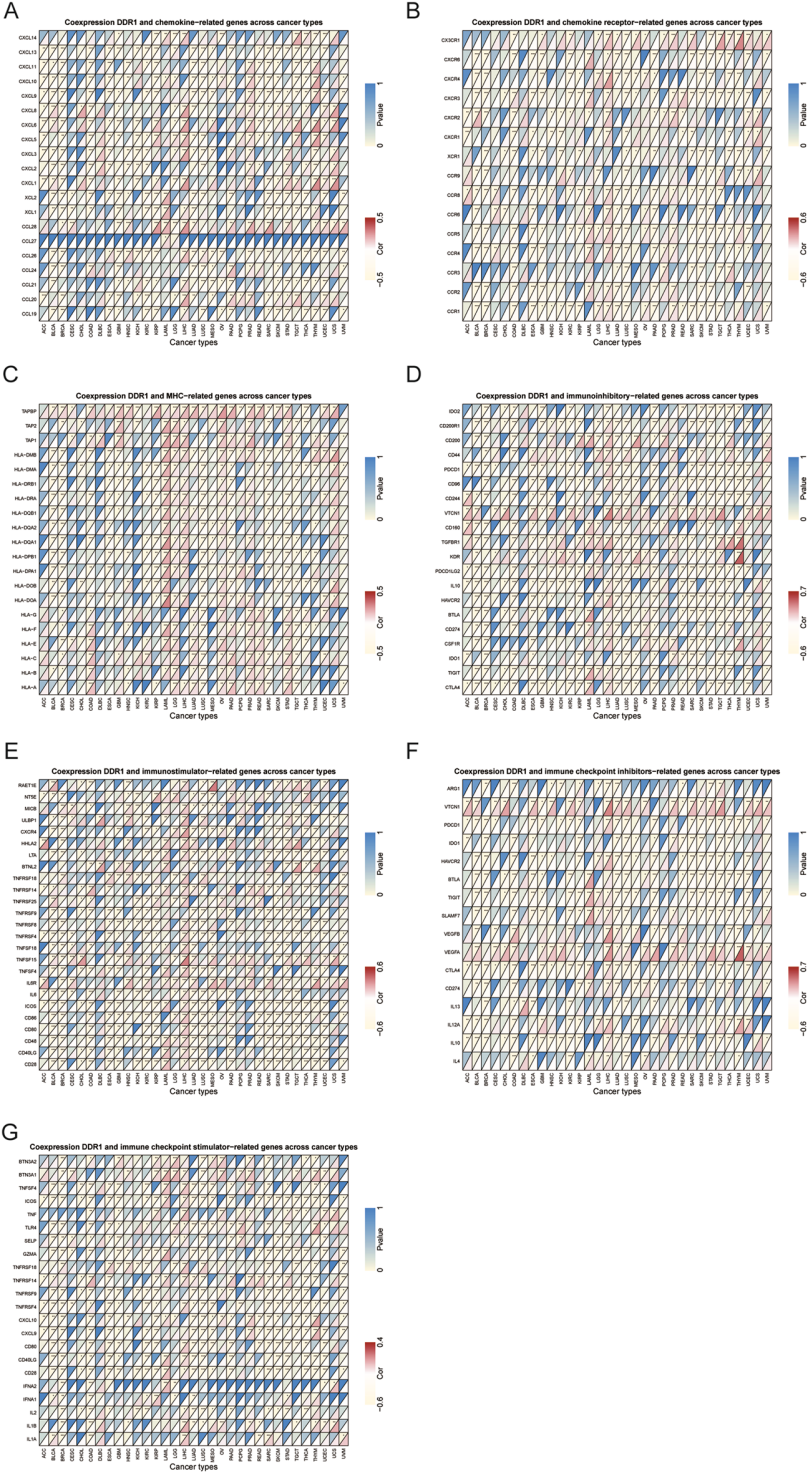
Co-expression of DDR1 and immune-related genes. *P < 0.05, **P < 0.01, ***P < 0.001.

We next conducted gene co-expression analyses to explore the relationship between DDR1 expression and immune-related genes in 33 tumors, including chemokine, chemokine receptor, MHC, immunostimulatory, immunosuppressive, and immune checkpoint-related genes. Our data showed that CXCL10 (chemokine-related gene) and CXCR3 (chemokine receptor-related gene) were negatively correlated with DDR1 expression in 11 tumors, except for LAML and PRAD (Fig. 9A, B). As shown in Fig. 9C, DDR1 expression was negatively correlated with TAPBP (MHC-related gene) in KICH, PCPG, SARC, and THCA, while positively correlated in other 19 cancers. In addition, we also analyzed the association between DDR1 and immunostimulatory, immunosuppressive, and immune checkpoint-related genes (Supplementary Table S1). The results illustrated that CTLA4 and ICOS were negatively correlated with DDR1 among 19 cancers while positively correlated with DDR1 in LAML and LIHC (Fig. 9D, E). Intriguingly, the expressions of TIGIT and CD28 were positively correlated with those of DDR1 in LAML and LIHC but negatively correlated with those of DDR1 expression in the other 17 cancers (Fig. 9F, G). These results reveal that DDR1 may promote tumor progression and immune escape by regulating immune-related genes.

### The prediction of the correlation between DDR1 expression and drug sensitivity

**Figure 10:**
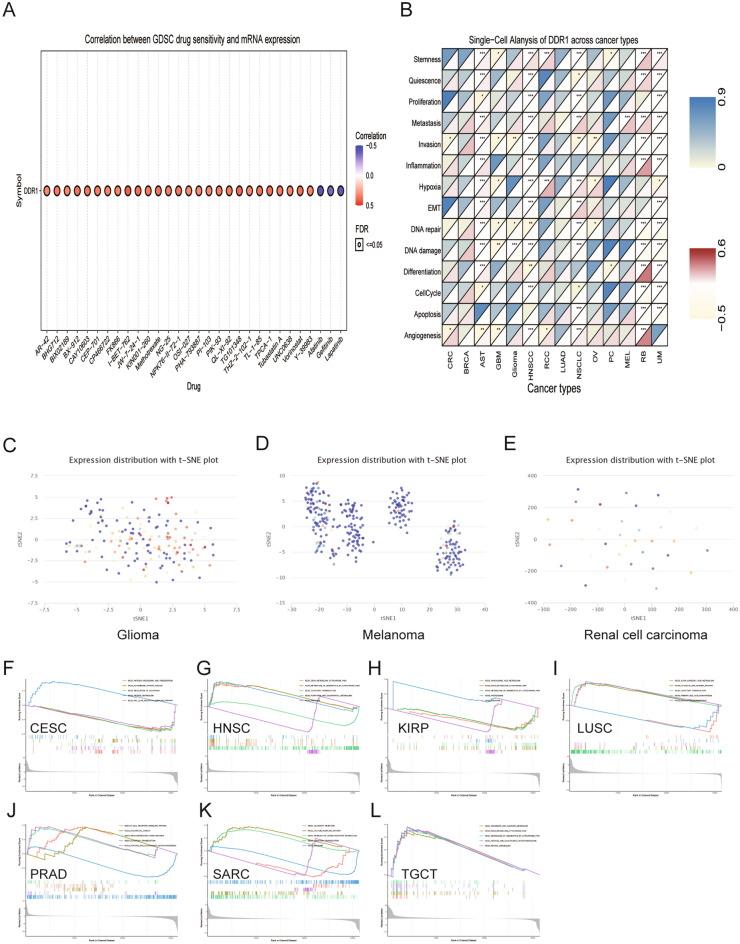
The relationship between DDR1 mRNA expression and drug sensitivity and tumor functional status and the results of GSEA analysis. (**A**) Heatmap illustrating the correlation between DDR1 expression and different tumor functional statuses based on the CancerSEA database. *P < 0.05, **P < 0.01, ***P < 0.001. (**B**) Figure summarizes the correlation between DDR1 expression and the top 30 sensitivity drugs across cancers based on the GDSC database. (**C–E**) T-SNE diagram demonstrates DDR1 expression profiles in single cells of glioma, melanoma, and renal cell carcinoma samples. (**F–L**) KEGG pathway analysis of DDR1 in multiple cancers. Curves of different colors show different functions or pathways regulated in different cancers. Peaks on the upward curve indicate positive regulation and peaks on the downward curve indicate negative regulation.

The association between the anticancer drug sensitivity and DDR1 mRNA expression was determined through the GDSC database, and we found that DDR1 expression was significantly correlated with the responses to 151 drugs. Our data showed that DDR1 expression was positively correlated with the sensitivity to the following drugs in most cancers; AR-42, I-BET-762, TG101348, JW-7-24-1, TPCA-1, vorinostat, methotrexate, TL-1-85, BX-912, and NPK76-II-72-1 (Fig. 10A, Supplementary Table S1). In contrast, DDR1 expression was negatively correlated with the sensitivity to three drugs or small molecules, including afatinib, gefitinib, and lapatinib. These results indicate that DDR1 is a potential therapeutic target in cancers.

### Expression pattern of DDR1 in single-cell and its relationship with cancer functional status

Single-cell transcriptomic sequencing is a crucial technique for analyzing diverse cancer cells, immune cells, endothelial cells, and stromal cells^20^. To verify DDR1 expression and its relationship with tumor functional status at the single-cell level in different cancers, using the CancerSEA database, we found that DDR1 expression was correlated with angiogenesis, DNA damage, hypoxia, stemness, metastasis, and DNA repair in most cancers (Fig. 10B). For instance, DDR1 expression was positively correlated with hypoxia, stemness, and angiogenesis in renal cell carcinoma (RCC) and metastasis in melanoma (MEL), while negatively correlated with DNA damage in glioma. DDR1 expression profiles were shown in single cells of glioma, MEL, and RCC by a t-SNE diagram (Fig. 10C–E). These results demonstrate that DDR1 participates in tumor development and metastasis.

### Gene set enrichment analysis of DDR1

Next, we evaluated the pathway through which DDR1 may involve by using GSEA analysis, in 33 tumor types from TCGA. Our data indicated that DDR1 was positively correlated with drug metabolism: cytochrome P450 and metabolism of xenobiotics by cytochrome P450 pathway in HNSC and testicular germ cell tumors (TGCT), while negatively correlated in KIRP (Fig. 10G, H, L). As shown in Fig. 10F, I, DDR1 was negatively associated with the regulation of autophagy in CESC while positively in LUSC. In addition, DDR1 was predicted to be a negative regulator of the ribosome and olfactory transduction in HNSC and SARC. In summary, these results suggest that DDR1 may serve as a potential therapeutic target in cancers.

## Discussion

The pan-cancer analysis provides a comprehensive understanding of the molecular aberrations and functional roles across various cancers. It helps to find new diagnostic biomarkers and new therapeutic targets for cancers^21,22^. Disk-like domain receptor 1 (DDR1) is a tyrosine kinase receptor that binds specifically to and is activated by collagen^5^. Accumulating evidence indicates that DDR1 is closely related to the occurrence and development of various tumors^8–10^. However, DDR1 has not thoroughly studied in cancer, and its role in tumorigenesis or pan-cancer is still unclear. Therefore, we conducted a pan-cancer analysis of DDR1 in 33 different cancers based on the data from the most comprehensive databases also explored the expression of DDR1 in ccRCC, BCa, and PCa cells for the first time.

We first assessed the expression and prognostic significance of DDR1 across cancers. Our results showed that DDR1 was dysregulated in 17 types of cancer, which was consistent with previous studies^11,12,23–27^. Our in vitro results were also consistent with the results of bioinformatics analysis. In contrast, our research reached the opposite conclusion in cervical cancer^13^. For example, DDR1 is highly expressed in GBM and leads to the resistance of tumor cells to radiotherapy and chemotherapy by affecting the Akt and mTOR signaling pathways^11^. Inhibition of DDR1 activation attenuated the proliferation and migration of HNSCC cells and increased the response to cisplatin^23^. Another study reported that DDR1 promoted cell proliferation and migration of thyroid cancer cells by regulating insulin receptor isoform-A (IR-A)^26^. Moreover, silencing of DDR1 increased cisplatin sensitivity in LUAD cells with KRAS mutations^27^. DDR1 promoted the intrahepatic metastasis of colon cancer by interacting with liver stromal cells to regulate liver stromal remodeling^28^. Regarding cervical cancer, our results challenge previous research, which indicated that metformin inhibited the proliferation of cervical cancer cells by upregulating the expression of DDR1^13^. This discrepancy may be due to differences in tumor cells, or the lack of a rescue experiment in the previous research. Notably, the overexpression of DDR1 was correlated with a better prognosis in KIRC, MESO, and UVM but was the opposite in COAD and UCS. Supporting that, DDR1 downregulation was found in ccRCC and related to shorter OS^14^. These findings demonstrate that DDR1 can be used as a biomarker of prognosis for various cancers.

Both DNA methylation alterations and RNA methylation modification play a crucial role in tumorigenesis^15,16^. Our study showed that DDR1 expression was significantly correlated with DNA methylation in eight tumors (positively correlated in BRCA, KIRP, LIHC, LUAD, LUSC, THCA, and UCEC, but negatively correlated in KIRC). Furthermore, DDR1 expression was positively correlated with the expression of m6A-, m5C-, and m1A-related genes in most cancers, including HNSC, BLCA, LIHC, and THCA. These findings suggest that the change in the epigenetic status of DDR1 may contribute to tumorigenesis. Tumors with defects in the MMR system will cause high levels of MSI, which leads to the aggravation of TMB and results in tumor occurrence^18^. Our results illustrated that DDR1 expression was highly correlated with MMR gene expression, MSI, and TMB in most cancers. These results indicate the function of DDR1 in mediating tumorigenesis by regulating DNA and RNA methylation, MMR gene expression, MSI, and TMB, which was consistent with previous studies^15,16,18^.

Our results show that DDR1 also plays a vital role in cancer immunity. TME plays a decisive role in tumor initiation, progression, and the response to therapies^19^. According to ESTIMATE scores, there were negative correlations between DDR1 expression and stromal and immune cell content in the TME of 14 cancers, which was supported by a previous study^28^. Tumor-infiltrating immune cells made a vast contribution to the homeostasis of TME and played a vital role in the occurrence, development, and immunotherapy response of tumors^29^. Our data revealed that DDR1 expression was negatively correlated with M0 macrophages and activated CD4 memory T cells in most cancers. Supported that, DDR1 overexpression was found in breast cancer and inhibited T cell infiltration^10^. Furthermore, our study also determined the co-expression of DDR1 with immune-related genes, including chemokines, chemokine receptors, MHC, immunostimulatory-related genes, immunosuppressive-related genes, and immune checkpoint-related genes. DDR1 expression was negatively correlated with most immune-related genes, which contribute to tumor development but was positively correlated with TAPBP^30,31^. In all, these results indicate that regulation of DDR1 expression may be a promising strategy to increase the efficacy of immunotherapy.

Mounting evidence has demonstrated that some new advances have been made in cancer treatment, such as RNA interference^32^, targeting the ubiquitin–proteasome pathway^33^, and targeting tumor suppressor genes^34^. However, drug resistance is also a major obstacle in pre-clinical and clinical therapeutics. We analyzed the correlation between DDR1 expression and IC_50_ of over 750 anti-cancer drugs. The results suggested that high DDR1 expression could reduce the sensitivity of many drugs but enhance the sensitivity of afatinib, gefitinib, and lapatinib, indicating its potential role in drug resistance. This finding reveals that regulating the expression of DDR1 can also be a promising strategy to enhance the efficacy of the anti-cancer drugs, supported by previous research^11,23,27^.

Finally, we used the CancerSEA database and GSEA analysis to address the specific function of DDR1 across different cancers. The results showed that DDR1 was closely related to angiogenesis, DNA damage, hypoxia, stemness, and metastasis at the single-cell level in most cancers. Consistent with our data, DDR1 is reported to be involved in angiogenesis^35^, DNA damage^36^, hypoxia^37^, stemness^9,11,26^, and metastasis^28^ and thus is involved in cancer development. The KEGG pathway analysis demonstrated that DDR1 expression was significantly correlated with pathway of drug metabolism, regulation of autophagy, ribosome, and olfactory transduction. Supporting these results, DDR1 has been reported to play a vital role in drug metabolism^38^ and autophagy^11^, leading to tumor progression.

Our study has several limitations. First, some contradictory findings of individual cancers in our study were observed. Therefore, it is necessary to further investigate the expression and function of DDR1 using a large sample size. Second, our findings suggest that DDR1 can be serve as a prognostic factor for different tumors, which needs further verification. Third, the effects of DDR1 on the tumor microenvironment and immunotherapy require experimental and clinical validation. Last, the current study was conducted mostly based on bioinformatic analysis and in vitro studies; further analysis of clinical samples from cancer patients are necessary to confirm the aberrant expression of DDR1.

## Conclusions

In summary, our first pan-cancer analyses of DDR1 indicate that this factor is differentially expressed between tumor tissues and normal tissues and reveals a correlation of DDR1 with DNA methylation and RNA methylation-related genes. Our findings suggest that DDR1 can serve as a prognostic factor for different tumors. Moreover, DDR1 expression is associated with the expression of MMR gene and with MSI, TMB, and immune cell infiltration across different cancer types. Its impact on tumor immunity and drug sensitivity also varies with tumor type. Importantly, in vitro experiments confirmed our bioinformatics analysis results in ccRCC, BCa, and PCa cell lines. These findings may help clarify the role of DDR1 in tumorigenesis and development and provide a reference for more accurate and personalized immunotherapy in the future.

## Supplementary Information

Supplementary Figure S1.

Supplementary Figure S2.

Supplementary Table S1.

## Data Availability

The datasets are available from TCGA database (http://cancergemome.nih.gov/) and GTEx database (http://gtexportal.org/home/). The original contributions presented in the study are included in the article/Supplementary Material. Further inquiries can be directed to the corresponding authors.
