# Supplementary figures and images for: A pan-cancer analysis of DDR1 in prognostic signature and tumor immunity, drug resistance
Source: Sci Rep. 2023 Apr 8;13:5779. doi: 10.1038/s41598-023-27975-9 (PMC10082773; doi:10.1038/s41598-023-27975-9)

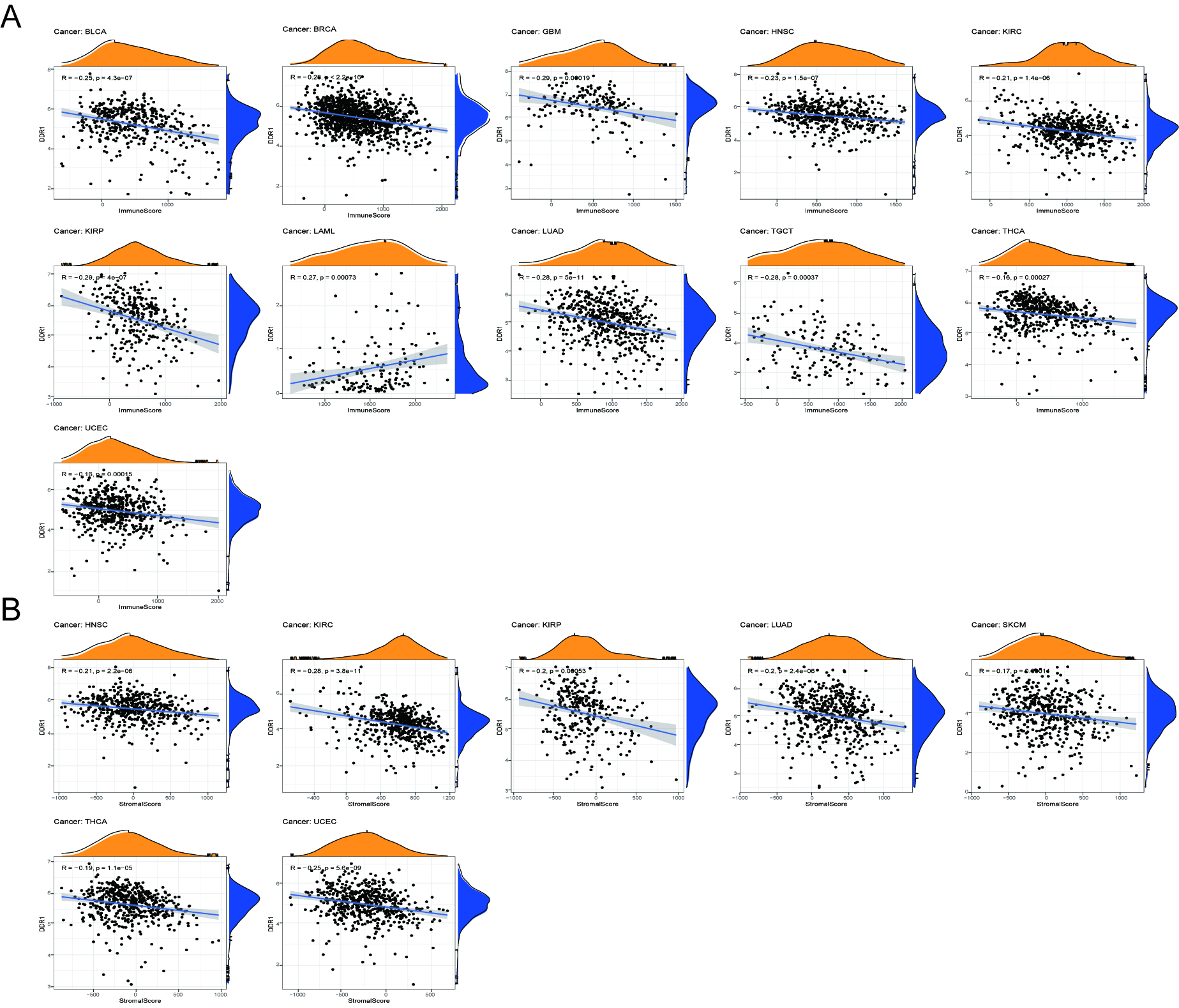

Supplement: Supplementary file 1 — Supplementary Figure S1. [file 41598_2023_27975_MOESM1_ESM.tif]

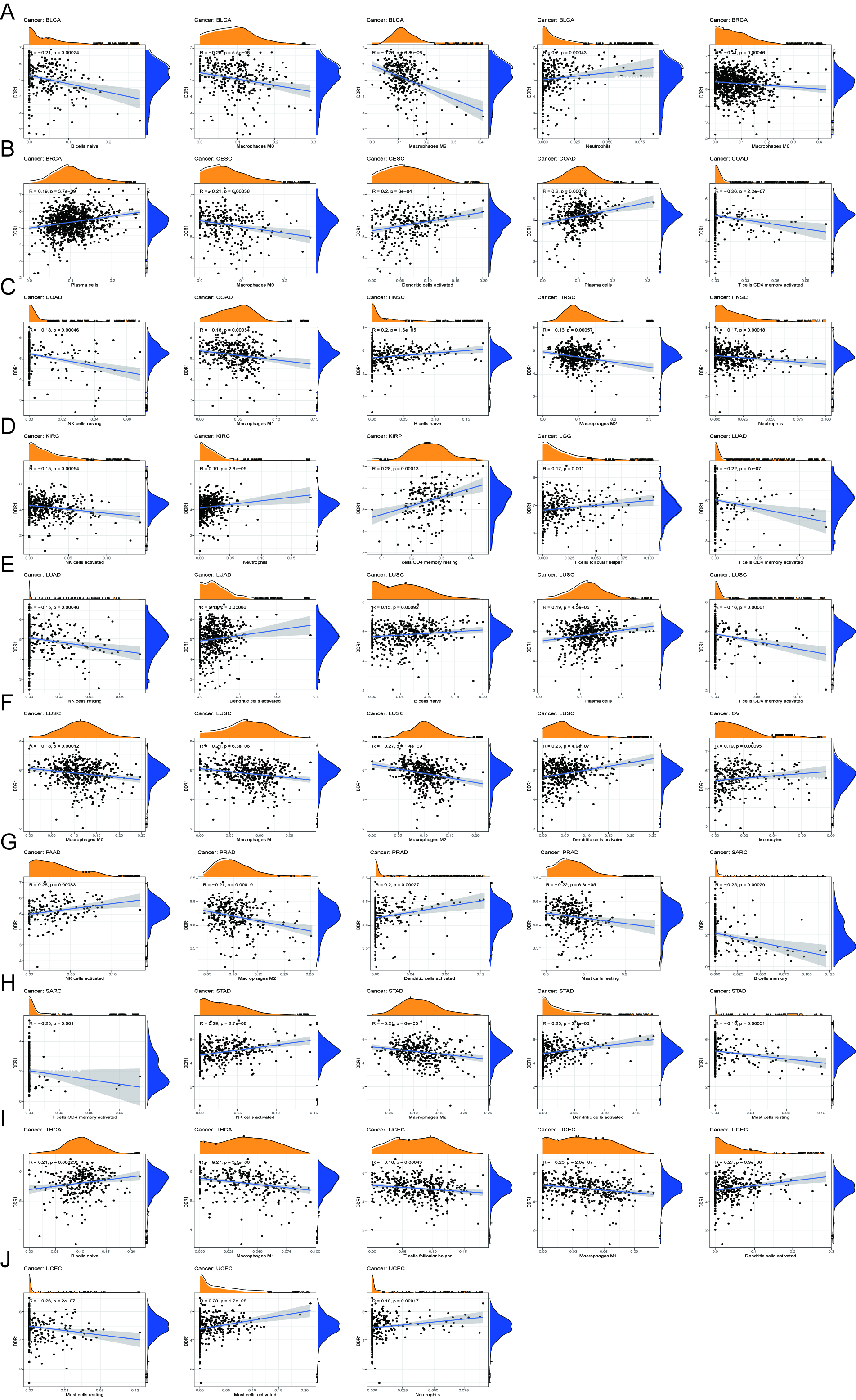

Supplement: Supplementary file 2 — Supplementary Figure S2. [file 41598_2023_27975_MOESM2_ESM.tif]
